# Health-care utilization and associated factors in Gauteng province, South Africa

**DOI:** 10.1080/16549716.2017.1305765

**Published:** 2017-06-02

**Authors:** Admas Abera Abaerei, Jabulani Ncayiyana, Jonathan Levin

**Affiliations:** ^a^ Faculty of Health Sciences, School of Public Health, Division of Epidemiology and Biostatistics, University of the Witwatersrand, Johannesburg, South Africa; ^b^ College of Health and Medical Sciences, School of Public Health, Haramaya University, Harar, Ethiopia

**Keywords:** Health-care, health-care utilization, immigrants, South Africa

## Abstract

**Background**: More than a billion people, mainly in low- and middle-income countries, are unable to access needed health-care services for a variety of reasons. Possible factors influencing health-care utilization include socio-demographic and economic factors such as age, sex, education, employment and income. However, different studies have showed mixed results. Moreover, there are limited studies on health-care utilization.

**Objective**: This study aimed to determine health-care utilization and associated factors among all residents aged 18 or over in Gauteng province, South Africa.

**Methods**: A cross-sectional study was conducted from data collected for a Quality of Life survey which was carried out by Gauteng City-Region Observatory in 2013. Simple random sampling was used to select participants. A total of 27,490 participants have been interviewed. Data were collected via a digital data collection instrument using an open source system called Formhub. Coarsened Exact Matching (CEM) was used to improve estimation of causal effects. Stepwise multiple logistic regression was employed to identify factors associated with health-care utilization.

**Results**: Around 95.7% reported usually utilizing health-care services while the other 4.3% reported not having sought health-care services of any type. Around 75% of participants reported reduced quality of public health services as a major reason not to visit them. Higher odds of reported health-care utilization were associated with being female (OR = 2.18, 95% CI: 1.88–2.53; *p* < 0.001), being White compared to being African (OR = 2.28, 95% CI: 1.84–2.74; *p* < 0.001), and having medical insurance (OR = 5.41, 95% CI: 4.06–7.23; *p* < 0.001). Lower odds of seeking health-care were associated with being an immigrant (OR = 0.61, 95% CI: 0.53–0.70; *p* < 0.001).

**Conclusions**: The results indicated that there is a need to improve the quality of public health-care services and perception towards them as improved health-care quality increases the choice of health-care providers.

## Background

More than a billion people, mainly in low- and middle-income countries (LMICs), are unable to access needed health-care services for a variety of reasons [1] Utilization of health-care services, public or private, depends on socio-economic factors, cultural beliefs and practices, and most importantly the health system itself [2] Immigration status, distance from health-care services, availability, affordability and quality of health-care are also other important determinants that influence health-care utilization [3,4].

Ward, Mertens and Thomas [5] defined health seeking as any remedial actions that are undertaken by individuals to rectify perceived ill health for the purpose of finding appropriate interventions. Health seeking behaviour is commonly thought of as the ways in which people behave in relation to their health. It can also be thought of as the utilization of health-care services, which is an endpoint of the process of seeking care. In the present study, health-care seeking behaviour is measured by the utilization of health-care services, rather than other ways in which people behave in relation to their health.

The South African constitution guarantees health-care access for all, yet considerable inequalities remain, for a variety of reasons [6,7] The South African government is concerned that the policy changes initiated since 1994 have not yet resulted in the expected outcomes; for example utilization levels remain relatively low in the public health-care sector. There has been little monitoring and evaluation of the impact of the new policies on the access by individuals of a lower socio-economic status to health-care [8].

Traditionally infectious diseases have a higher prevalence among individuals with a lower socio-economic status. While this is still largely the case, the increasing complexity of health problems has led to infectious diseases now also occurring amongst individuals with a higher socio-economic status. Similarly, chronic diseases, previously thought to be most prevalent among individuals of a higher socio-economic status, are now also occurring among individuals of a lower socio-economic status. As a result of the ongoing urbanization in South Africa, coupled with the slow growth of the economy, the relationship between socio-economic status and health status is becoming more complex [6].

Moreover it is important to quantify the degree to which individuals who are not well seek care from public health-care facilities, private health-care facilities or traditional healers, or to establish whether they engage in self-treatment from pharmacies. For example in designing an anti-retroviral therapy (ART) delivery programme, the adherence to the programme could depend on the ways in which individuals engage with the health-care system [9,10].

Immigrants, as a group of people who are at increased risk of poor physical, psychological and social health outcomes and also at risk of receiving sub-optimal health-care, constitute a vulnerable population [11] There is thus a need to understand the health-care needs of this group, as well as their perceptions of the health-care system. In doing so one must be aware of the challenges due to the heterogeneity of this group and also the particular challenges posed by illegal immigrants [11].

Knowledge about health-care seeking behaviour can help in developing or modifying health-care policies, in order to identify possible difficulties and if necessary to develop appropriate interventions. South Africa is currently developing a National Health Insurance (NHI) plan. The aim of the NHI will be to provide universal access to health-care. Information on the health-care seeking behaviour of individuals and also on the perceptions of the health services will help the NHI to achieve its goals.

In addition to limited existing studies conducted on health-care utilization in South Africa, this study covers a very large area with sufficient sample size and a host of important variables that will help in explaining the health-care utilization patterns. Therefore this study aimed to determine health-care utilization and factors affecting it among all residents of Gauteng province, South Africa.

## Methods

### Study sample and data collection

A secondary data analysis of data from the Quality of Life Survey (QoL) was carried out by Gauteng City-Region Observatory (GCRO). The survey measures a wide range of variables including socio-demographic variables, quality of life and attitudes towards the health-care services. Study participants were selected from Gauteng province, South Africa in 2013. Gauteng is the province with the largest population, estimated to be 12,272,263 (Census 2011), despite having the smallest area; thus it has the highest population density in South Africa of 675 people per km^2^.

The study population consists of all people residing permanently in Gauteng province who were aged 18 or older in 2013. Simple random sampling was employed to select the samples. Gauteng province consists of 10 municipalities and it is subdivided into 508 wards. Within these wards there are Small Area Levels (SALs) which were derived from the Population Census Enumerator Area (EA) polygons. SAL codes and geography were derived from the Statistics South Africa information (Census 2011). The simple random sampling method was used to select the SALs from each ward, then the minimum numbers of interviews for each ward were 30 and 60 interviews for those falling in district municipalities and metropolitan municipalities, respectively. The end result was that across the 508 wards, 26,387 successful interviews had to be completed and these interviews were distributed across 16,400 SALs out of a total of 17,840 SALs. The ‘NEXT’ birthday method was used to select the respondents from the selected households [12].

### Data collection

In the primary research, data were collected via a digital data collection instrument using an open source system called Formhub and administered on a tablet device. Once a questionnaire was administered in the field, it was uploaded using Internet connectivity to a cloud server from where it could be accessed and downloaded online. Approximately 120 of these devices were used in the field.

### Variables and measurements

The main outcome variable for this study was a binary variable measuring health-care utilization, coded as 1 (or ‘sought care’) if the participant obtained care from public or private health facilities and as 0 (or ‘did not seek care’) if the participant went to a traditional healer or didn’t visit any health-care provider. The explanatory variables included age, sex, population group, immigration status, place of residence, education level, whether or not the participant had medical insurance, employment status, household income, distance from public transport, and the perception of accessibility, affordability and quality of the health-care services.

Information collected included demographic and socio-economic variables: age (continuous variable); sex; population group (African, White, Coloured and Indian/Asian); educational level (‘no formal education’, ‘primary’, ‘secondary’ and ‘tertiary and above’); employment status (‘employed’, ‘unemployed’ and ‘others’); household-income (lower, middle and upper class); immigration status (South African and immigrant); satisfaction about standard of living (very satisfied/satisfied, neutral, dissatisfied/very dissatisfied); satisfaction towards health-care services that the government provides; medical insurance (medical aid, hospital plan and no insurance); and reasons for not using health-care services (distance/accessibility, availability, cost, quality of care and other cultural factors).

Health-care utilization was measured by means of two questions. The first asked whether or not the participant sought health-care, while the second asked where the participant usually went for health-care. In our study, participants who answered that they did seek health-care and went to private facilities, public facilities or both private and public facilities were regarded as seeking health-care, while participants who did not seek health-care or who went to traditional healers were regarded as not seeking health-care.

### Data analysis

Educational status was categorized into ‘No formal education’, grades 1–8 ‘Primary’, grades 9–12 ‘Secondary’ and above grade 12 ‘Tertiary and above’. Total household income was categorized into ‘Lower income’ (< 6400 Rand [R] per month), ‘Middle income’ (R6400 – R51,200 per month) and ‘Upper income’ (> R51,200 per month). Medical insurance was categorized into ‘medically insured’ for participants with either medical aid or a hospital plan and ‘medically not insured’ for participants without any of these.

Descriptive analysis was performed to describe the health-care utilization behaviour of the community and types of health-care services utilized by socio-demographic characteristics of study participants using proportions. Associations with socio-demographic variables were assessed using the chi-square test.

A crude comparison of the proportions seeking health-care between groups ignores the influence of other characteristics on health-care utilization behaviour. These factors include age, sex, educational status, household income level and employment status. In order to take these factors into account we employed a type of matching known as Coarsened Exact Matching (CEM) in the analysis. CEM [13] has previously been used in the analysis of health service utilization [10].

In general, matching is a method of controlling for the confounding effect of additional variables in the analysis of observational data. In matching, observations are removed from the analysis data-set so that the ‘treated’ and ‘control’ groups are balanced on additional variables [14,15] Two such methods of matching are propensity score matching (PSM) and exact matching (EM). The CEM differs from these methods in that it compares observations from the treatment and control groups that are only approximately similar. This gives CEM the advantage of retaining more observations for analysis than PSM and EM, as well as ensuring robustness to measurement errors and requiring fewer assumptions, in addition to being very fast computationally [13].

The motivation for using CEM is that while PSM and EM provide perfect balance, a large proportion of observations will be lost as the number of covariates on which the matching is based increases, and hence few matches will be produced. Let us consider a sample of size n drawn from a population of size N, where n < N. Suppose T_i_ (one of the covariates) is regarded as the ‘treatment received’ and is coded as T_i_ = 1 if individual i receives the treatment and as T_i_ = 0 if individual i is a control (i.e. does not receive the treatment). Let Y be the outcome variable so Y_i_ (1) is the outcome for an individual who receives the treatment and Y_i_ (0) is the outcome for a control individual. Hence an outcome can be written as:

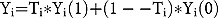

Note that Y_i_ (0), which is the potential outcome for individuals who do not receive treatment (i.e. controls), is unobserved if individual i receives treatment and similarly Y_i_ (1), the potential outcome for individuals who receive treatment, is unobserved if the individual is a control [13].

In our study, the dichotomous variable presence or absence of medical insurance was used as a treatment variable to carry out the matching as it is an important factor that determines whether an individual seeks health-care or not. Thus the treatment cases are medically insured individuals and the non-treatment controls are medically non-insured individuals.

The conceptual framework used for analysis was the health-care model originally proposed by Andersen [16] as modified by Ahmed in 2005 [17] In this model factors which influence health-care utilization consist of predisposing factors (age, sex, education, ethnicity, religion, occupation, employment, knowledge and previous experience), enabling factors (household poverty, perceived illness, out-of-pocket expenditure, availability, affordability and quality of health-care services and medical insurance) and need factors (perceived severity, help from peers etc.). Using this model as the basis for analysis, the pre-treatment variables used for CEM include (i) age (grouped), (ii) sex, (iii) population group (African, White, Coloured and Indian/Asian), (iv) educational status (none, primary, secondary or further), (v) employment status, (vi) place of residence, (vii) immigration status (born in South Africa or immigrant), (viii) satisfaction with the health-care services provided by the government and (ix) self-perceived satisfaction with one’s standard of living (satisfied, neutral, dissatisfied).

The CEM was carried out using the covariates previously specified and using the default automatic binning algorithm. This resulted in 26,318 observations being matched and 1172 observations being dropped. The quality of the matched data was assessed by comparing imbalances found before and after matching. In our case there was a substantial reduction in all of the covariates, not only in the means, but also in the quartiles and marginal distributions, with most imbalances being reduced to zero after matching. Hence we can conclude that the matching was successful. The matching algorithm generated weights for use in logistic regression models fitted to the original data.

After carrying out the CEM, multiple logistic regression models were fitted to find factors associated with health-care utilization. The candidate variables considered were age, employment status, sex, population group, whether or not the individual had medical insurance, educational status, immigration status, total household income, place of residence and self-perceived satisfaction with the standard of living.

Firstly all variables were screened by carrying out univariable analyses using a liberal *p*-value of 0.10. All variables significant at the 10% level were included in the multivariable analysis, and then backward elimination was used to remove variables not significant at the 5% level. The model was refitted using forward selection – in this case the two methods resulted in the same model. Interactions between explanatory variables were examined on a pairwise basis. The goodness of fit of the final model was investigated using the Hosmer and Lemeshow goodness of fit test. All analyses were carried out using Stata release 13.1 (Stata Corporation, College Station, Texas, USA).

## Results

### Socio-demographic characteristics of the study sample

Of the total population of 27,490 interviewed, 15,655 (57%) were females and the mean age was 39.4 ± 15.4 years (Table 1). The majority of the study participants were African by population group – 23,059 (84%) – and around 8887 (32%) reside in the city of Johannesburg. Data on educational status showed that most participants 16,044 (59%) had secondary education while only 585 (2%) had no formal education. More than half of participants were unemployed 15,815 (58%) and most participants – 21,458 (78%) – had no medical insurance. Around 16,224 (59%) had a total household income which falls under ‘lower class’ (lower class was defined in this study as families with a total household income of less than R6,400 per month), while only 1.3% fall under ‘upper class’, which was defined as those who have a total household income of more than R51,200 per month. Sixty-two per cent responded that they were satisfied with their standard of living. Among those using government health-care services, 8826 (37%) of the study participants were either unsatisfied or very unsatisfied with the health-care services that the government provides.

**Table 1.** Baseline characteristics of study participants by health-care utilization in Gauteng province, South Africa, 2013.

Characteristics	Category	Sought health-care [n = 26,316]	Did not seek health-care [n = 1174]	χ^2^ value, *p*-value	Total [n = 27,490]
**Age** (mean ± SD)		39.9 ± 15.4	34.7 ± 13.2	–	39.4 ± 15.4
**Sex**	Male	11,017 (93.1%)	818 (6.9%)	219.1, < 0.001	11,835 (43.1%)
	Female	15,299 (97.7%)	356 (2.3%)		15,655 (56.9%)
**Population group**	African	21,969 (95.3%)	1090 (4.7%)	73.7, < 0.001	23,059 (83.9%)
	White	2893 (98.8%)	33 (1.2%)		2926 (10.6%)
	Coloured	876 (98.0%)	18 (2.0%)		894 (3.3%)
	Indian/Asian	484 (96.6%)	17 (3.4%)		501 (1.8%)
**Level of education**	No formal	557 (95.2%)	28 (4.8%)	26.9, < 0.001	585 (2.1%)
	Primary	5022 (95.9%)	214 (4.1%)		5236 (19.0%)
	Secondary	15,326 (95.5%)	718 (4.5%)		16,044 (58.4%)
	Tertiary	5110 (96.4%)	183 (3.6%)		5293 (19.3%)
**Municipality**	Johannesburg	8491 (95.5%)	396 (4.5%)	10.8, 0.46	8887 (32.3%)
	Tshwane	5976 (95.2%)	302 (4.8%)		6278 (22.8%)
	Emfuleni	1385 (96.3%)	54 (3.8%)		1439 (5.2%)
	Others	4116 (95.9%)	178 (4.1%)		4294 (39.7%)
**Employment status**	Employed	11,094 (95.0%)	581 (5.0%)	27.9, < 0.001	11,675 (42.5%)
	Unemployed	15,222 (96.3%)	593 (3.7%)		15,815 (57.5%)
**Total income**	Lower class	15,522 (95.7%)	702 (4.3%)	31.5, 0.01	16,224 (59.0%)
	Middle class	3866 (97.4%)	103 (2.6%)		3969 (14.4%)
	Upper class	355 (97.8%)	8 (2.2%)		363 (1.3%)
	Refusal	6573 (94.8%)	361 (5.2%)		6934 (25.2%)
**Self-perceived satisfaction**	Satisfied	16,441 (95.7%)	715 (4.3%)	12.8, 0.04	17,156 (62.4%)
**of living standard**	Neutral	2232 (96.2%)	88 (3.8%)		2320 (8.4%)
	Unsatisfied	7643 (95.1%)	371 (4.9%)		8014 (29.2%)
**Satisfaction on government**	Satisfied	11,815 (98.3%)	201 (1.7%)	11.3, 0.21	12,016 (43.7%)
**health-care**	Neutral	2614 (95.8%)	115 (4.2%)		2729 (9.9%)
	Unsatisfied	8645 (97.9%)	181 (2.1%)		8826 (32.1%)
**Migration status**	South African	14,879 (97.1%)	438 (2.9%)	73.2, < 0.001	15,317 (55.7%)
	Immigrant	11,437 (93.9%)	736 (6.1%)		12,173 (44.3%)
**Medical insurance**	Yes	5977 (99.1%)	55 (0.9%)	169.6, < 0.001	6032 (21.9%)
	No	20,339 (94.7%)	1119 (5.3%)		21,458 (78.1%)

### Socio-demographic characteristics by health-care sought

Overall only 1174 (4.3%) of 27,490 respondents did not seek health-care. Males (6.9%) were more likely than females (2.3%) to not seek health-care. As the education level increased, respondents were more likely to seek health-care. Those of upper and middle socio-economic class were more likely to seek health-care than those of lower class and those who refused to disclose their income. Respondents who were satisfied with government-provided health-care were more likely to seek health-care. Immigrants were less likely than those born in South Africa to seek health-care, while respondents with medical insurance were more likely to seek health-care, with over 99% of such respondents seeking health-care.

### Types of health-care sought by socio-demographic characteristics

When asked about usual place of health-care services, 65.4% of participants usually used public health-care facilities; around 24% usually used private health-care facilities; 0.5% usually went to traditional healers; and 3.8% did not visit any type of health-care services when they needed health-care (Table 2). Among those who visited private health-care services, men visited more frequently than women (28.8% vs. 21.4%); Whites visited more frequently than Africans, Coloured and Indian/Asians (79.8% vs. 16.1%, 32.0% and 64.1%, respectively); and those with medical insurance visited more frequently than those without any medical insurance (82.1% vs. 8.1%).

**Table 2.** Baseline characteristics of study population by type of health-care used in Gauteng province, South Africa, 2013.

Predictors	Private health-care facilities (n = 6691)	Public health-care facilities (n = 17,978)	Use public and private facilities (n = 1647)	Traditional healers (n = 141)	No health-care (n = 1033)
**Overall**	6691 (24.3%)	17,978 (65.4%)	1647(6.0%)	141 (0.5%)	1033 (3.8%)
**Age**	41.4 ± 15.4	39.4 ± 15.4	40.0 ± 15.0	39.0 ± 12.8	34.2 ± 13.1
**Sex**					
Male	3407 (28.8%)	6868 (58.0%)	742 (6.3%)	86 (0.7%)	732 (6.2%)
Female	3284 (21.4%)	11,110 (71.0%)	905 (5.8%)	55 (0.4%)	301 (1.9%)
**Population group**					
African	3718 (16.1%)	16,869 (73.2%)	1382 (6.0%)	140 (0.6%)	950 (4.1%)
White	2334 (79.8%)	415 (14.2%)	144 (5.0%)	0	33 (1.1%)
Coloured	285 (32.0%)	506 (56.6%)	85 (9.5%)	0	18 (2.0%)
Indian/Asian	321 (64.1%)	134 (26.8%)	29 (6.0%)	0	17 (3.4%)
Others	33 (30.0%)	54 (50.0%)	7 (6.4%)	1 (0.9%)	15 (13.6%)
**Level of education**					
No formal	40 (6.8%)	497 (85.0%)	20 (3.4%)	8 (1.4%)	20 (3.4%)
Primary	381 (7.3%)	4391 (83.9%)	250 (4.8%)	39 (0.7%)	175 (3.3%)
Secondary	2989 (18.6%)	11,375 (71.0%)	962 (6.0%)	74 (10.5%)	644 (4.0%)
Tertiary	2650 (57.3%)	1418 (30.7%)	382 (8.3%)	14 (0.3%)	159 (3.4%)
Postgraduate	581 (86.7%)	60 (9.0%)	19 (2.8%)	1 (0.2%)	9 (1.3%)
Unspecified	50 (15.1%)	237 (71.4%)	14 (4.2%)	5 (1.5%)	26 (7.8%)
**Employment status**					
Unemployed	4111 (35.2%)	6103 (52.3%)	880 (7.5%)	62 (0.5%)	519 (4.5%)
Employed	2580 (16.3%)	11,875 (75.1%)	767 (4.9%)	79 (0.5%)	514 (3.3%)
**Medical insurance**					
Yes	4954 (82.1%)	538 (8.9%)	485 (8.0%)	4 (0.1%)	51 (0.85%)
No	1737 (8.1%)	17,440 (81.3%)	1162 (5.4%)	137 (0.6%)	982 (4.6%)

Almost all respondents who reported consulting traditional healers were from the African population group. Respondents with secondary education (10.5%) were more likely to consult traditional healers. Males (6.2%) were more likely to not use any type of health services than females (1.9%), as were those without medical insurance (4.6%) compared to those with medical insurance (0.85%).

**Figure 1.**
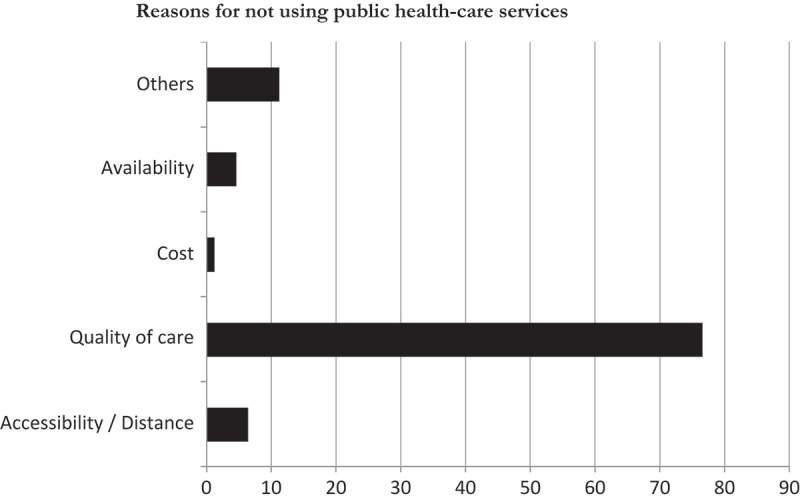
Reasons for not using public health-care services in Gauteng province, South Africa, 2013.

When asked about reasons for not using public health-care services, approximately 77% of participants reported quality of care at public health-care services as the main reason (Figure 1). Around 6.4% reported inaccessibility and 4.6% reported unavailability of public health-care services close to their residence.

### Factors associated with health-care utilization

For the multiple logistic regression the variables age, employment status, sex, population group, medical insurance, educational status, immigration status, total household income, place of residence and self-perceived satisfaction on standard of living were considered as candidate explanatory variables.

Stepwise logistic regression was used to select variables that should be included in the final parsimonious model. Total household income, place of residence, self-perceived satisfaction on standard of living, and satisfaction with health-care services the government provides were found not to be statistically significant. Age of participants, sex, population group, employment status, medical insurance and immigration status were found to be significantly associated with health-care utilization. The results of the final logistic regression are presented in Table 3. Higher odds of reported seeking health-care were associated with being female (odds ratio [OR] = 2.18, 95% confidence interval [CI]: 1.88–2.53; *p* < 0.001). Females are approximately twice as likely to seek health-care compared to males. Similarly, as age increases the odds of health-care utilization also increase (OR = 1.02, 95% CI: 1.01–1.03; *p* < 0.001), i.e. for a one-year increase in age the odds of seeking health-care increase by 2%.

**Table 3.** Stepwise logistic regression^1^ assessing factors associated with health-care utilization in Gauteng province, South Africa, 2013.

	Health-care utilization (N = 26,387)			
Predictors	Unmatched OR^2^	*p*-value	Adjusted OR^3^	*p*-value
**Age**	1.02 (1.015, 1.025)	< 0.001	1.02 (1.01, 1.03)	< 0.001
**Sex**				
Male	1		1	
Female	2.64 (2.31, 3.02)	< 0.001	2.18 (1.88, 2.53)	< 0.001
**Population group**				
African	1		1	
White	3.75 (2.65, 5.32)	< 0.001	2.28 (1.84, 2.74)	< 0.001
Coloured	2.09 (1.30, 3.34)	0.002	1.46 (1.13, 1.89)	0.004
Indian/Asian	1.55 (0.89, 2.70)	0.123	0.72 (0.57, 0.92)	0.009
Others	0.81 (0.25, 2.60)	0.730	1.38 (0.48, 3.91)	0.550
**Employment status**				
Unemployed	1		1	
Employed	0.79 (0.69, 0.90)	< 0.001	0.84 (0.72, 0.97)	0.020
**Medical insurance**				
No	1		1	
Yes	5.13 (3.90, 6.74)	< 0.001	5.41 (4.06, 7.23)	< 0.001
**Migration status**				
Born in S.A	1		1	
Immigrant	0.57 (0.50, 0.65)	< 0.001	0.61 (0.53, 0.70)	< 0.001

Notes: ^1^Hosmer and Lemeshow test *p*-value = 0.23; ^2^Unmatched OR is an estimate before CEM was done. ^3^Matched OR is after the data were matched using CEM.

CEM – Coarsened Exact Matching; OR – Odds Ratio.

Higher odds of reported health-care utilization were also associated with being White compared to being African (OR = 2.28, 95% CI: 1.84–2.74; *p* < 0.001); and with having medical insurance compared to not having any (OR = 5.41, 95% CI: 4.06–7.23; *p* < 0.001). In contrast, lower odds of seeking health-care were associated with being an immigrant compared to being a citizen of the Republic of South Africa (OR = 0.61, 95% CI: 0.53–0.70; *p* < 0.001); and being employed compared to being unemployed (OR = 0.84, 95% CI: 0.72–0.97; *p* = 0.02).

## Discussion and conclusion

### Discussion

The findings from this study provide important insights into health-care utilization from a population-based survey. The primary strength of this study is that it tries to assess predictors of general health-care utilization from a very large survey, which maximized the potential of the study to include vulnerable populations such as immigrants.

This study showed that a large proportion (95%) of study participants have usually sought health-care from either public or private health facilities when needed. South African legislation provides for universal health-care services, irrespective of ethnicity, socio-economic status and citizenship [18] This largely contributed to utilization of health services among the population. Previous studies in different settings [19,20] showed lower proportions of health-care utilization. This might be explained partly by previous studies having assessed health-care utilization within a specified time period (in the last 12 months, in the last 6 months etc.) while the current study assesses general health-care utilization when needing health services for any type of illnesses. Private health-care services were most commonly visited by the richest, medically insured and Whites.

In our study 0.5% of participants usually visited traditional healers when they were ill. This finding is consistent with a study done in Nepal [21] where 0.6% of participants visited traditional healers as their first choice. Another study in South Africa, however, showed that around half of adults were reported to have visited traditional healers prior to death [9] However, virtually all of these individuals also saw private or public health-care services. The discrepancy may be explained partly by the fact that while our study assessed where an individual usually seeks treatment, this study assessed whether an individual visited a traditional healer regardless of whether the individual had also visited other health-care services. The other possible explanation for the discrepancy could be due to the fact that Gauteng differs from other provinces of South Africa; it has a lower proportion of Africans, generally a higher level of education and a far lower proportion of rural population than the rest of South Africa.

Our study also showed differences between population groups in health-care seeking behaviour, with White and Coloured respondents being more likely to seek health-care than African respondents. This is consistent with a previous study which found differences between races in health seeking [22] This study showed sex differences with females being more likely to seek health-care than males. While previous studies [3,20] have similar findings, a few studies have suggested the opposite, with a study in Vietnam indicating that women are less likely to visit health services than men [23] These different findings might be due to the difference in the study settings and the difference in the years in which the studies were conducted. Over time, as the empowerment of women and the education level of women increase, the probability of women seeking health-care is likely to increase.

Education plays a crucial role in an individual’s decision to visit and utilize health-care services. In our study educational status did not seem to be a significant predictor of health-care utilization. However, previous studies indicated a positive association between education and health-care utilization [21,24–27] This discrepancy may be due to the fact that study participants in our study are 18+ years old and most of them had at least a secondary education.

In this study quality of care was reported by the majority (76%) of participants as a reason not to visit public health-care services. This reported reduced quality of care at public health-care services may be due to the high volume of patients in these places. However, there is a need for continued effort to improve the quality of public health-care services to increase health-care utilization by the people in the country. This finding is consistent with previous studies [22,27] where the majority of respondents indicated poor quality of care in public health-care services as a main reason not to visit them.

Lack of health insurance is a barrier to accessing health-care, especially for the poor and other disadvantaged groups. Our study unsurprisingly showed that insured individuals are more likely to visit health-care compared to uninsured individuals. This finding was consistent with previous studies done elsewhere [,^22^].

Understanding health-care utilization and perception towards health services of immigrant populations in countries like South Africa where there is a high proportion of immigrants is of great concern as it affects positive health outcomes. Our study showed that immigration status is significantly associated with health-care utilization in that immigrants are less likely to seek health-care services compared to those who were born in South Africa. This is in line with previous studies in the U.S [11] and Canada [28] where they indicated that immigrants have poor access to both medical services and public health services and programmes. The reasons put forward include legal status, i.e. fear of detection by authorities if they are undocumented, and other socio-economic barriers such as inability to afford health insurance [11].

### Strengths and limitations

Little research has been carried out on health-care utilization behaviour and access to health-care in South Africa. The primary strength of this study is that it assesses predictors of health-care utilization from a very large population-based representative sample survey, thereby maximizing the power of the study to detect significant associations. Secondly, the use of random sampling to select study participants improves both the internal and external validity of the study. Moreover, our study included migrant populations in which little is known about their health-care utilization. A wide variety of socio-economic and demographic factors that can influence the health-care utilization of an individual were also assessed in this study. The other strength is that, unlike other methods of matching such as PSM and EM, CEM does not require the data to be balanced in terms of pre-treatment variables in which case most of the data would be lost.

The primary data were collected to assess quality of life in Gauteng province, not to answer specifically the research question. As a result they miss some important health-related information. Whether the interviewee himself/herself was suffering from a specific health problem in the last 12 months and whether s/he sought health-care for the problem was not asked, as this reasonably gives a valid assessment of health-care utilization level rather than asking whether the participant generally seeks health-care. Specific types of health-care services such as maternal and children’s health-care utilization were also not assessed. In addition, data on cultural background, ethnicity and religion, which were not assessed in this study, could contribute useful information about health-care utilization.

Furthermore, since the research involved a sensitive topic, there is the possibility of bias since participants may be unwilling to disclose information that could pose a risk for themselves and for the group that they represent. For instance, in our study, immigration status was self-reported and if the participant is an illegal or undocumented migrant he or she might not report their correct status because of fear of detection by the authorities and the possible implications if they should be identified, which leads to underestimation of the number of immigrants found in the study area.

## Conclusions

This study indicated that the majority of study participants sought health-care for all types of illnesses and few respondents also reported traditional healers as a choice of health-care. Age and sex of participants, population group, immigration status and presence/absence of health insurance were significantly associated with health-care utilization.

The study showed that immigrants were less likely to utilize health-care compared to non-immigrants. This inequality in access to health-care indicates the need to incorporate immigrant populations in decision-making processes on various designs and implementations of health-care services at both the local and national levels. Health education and health promotion campaigns should focus on creating continuous awareness on chronic diseases and their risk factors.

Increased health service quality increases the choice of health-care providers relative to either going to traditional healers or self-treatment [25] Over three quarters of respondents mentioned poor quality of public health-care services as a reason not to visit them. This indicates the need to improve the quality of public health-care services and the perception towards them. This includes provision of medication and facilities, in-service training for health professionals on patient handling and service provision, and capacity building. Last but not least, there should be multi-sectoral collaboration in improving health-care access for vulnerable populations, those with lower socio-economic status and immigrants (both documented and undocumented).

## Paper context

Inequities in utilization of health-care persist among the South African population for various reasons. Research has shown controversial results on factors affecting health-care utilization. Most participants have used health-care services and a few either visited traditional healers or did not seek any type of health-care facilities. Immigrants, males and individuals with no health insurance are less likely to seek health-care. Promoting health service utilization, improving the quality of public health facilities and incorporating immigrant populations in decision-making processes are essential in order to enhance utilization of health services.
